# Advances in genetic and molecular mechanisms of crop resistance to stalk rot

**DOI:** 10.1007/s44154-025-00282-1

**Published:** 2026-01-07

**Authors:** Yueqi Kang, Mingxiu Ruan, Qinhan Hu, Zihao Gui, Jinyan Zhou, Jianbo Yao, Yuanyuan Cao, Ting Ding, Bo Wang, Fengquan Liu, Haiyang Jiang, Guichun Wu, Leiming Wu

**Affiliations:** 1 The National Engineering Laboratory of Crop Resistance Breeding, School of Life Sciences, Anhui Agricultural University, Hefei, 230036 China; 2 Anhui Province Key Laboratory of Crop Integrated Pest Management, Anhui Agricultural University, Hefei, 230036 China; 3 College of Plant Protection, South China Agricultural University, Guangzhou, Guangdong 510624 China; 4 Department of Plant Pathology, College of Agriculture, Guizhou University, Guiyang, 550025 China

**Keywords:** Fungal stalk rot, Bacterial stalk rot, Pathogenic mechanism, Resistant genes, Molecular regulation, Cell wall biosynthesis

## Abstract

As the global warming intensifies, along with increased planting density and straw retention practices, stalk rot (SR) has become one of major diseases that negatively impacts crop yield and quality. The distribution of SR pathogens, encompassing both fungal and bacterial agents, is significantly influenced by climate and agricultural factors. Although significant researches have been conducted on identifying fungal SR in different crop plants, there remains a lack of comprehensive reviews focused on the genetic and molecular mechanisms that contribute to crop resistance against fungal and bacterial SR. This review provides a comprehensive comparison of the pathogenic mechanisms associated with fungal and bacterial SR. It emphasized recently cloned genes and molecular regulations linked to resistance against SR, highlighted the pivotal role of several smart strategies in advancing gene discovery and functional research. Furthermore, it summarized the potential molecular regulatory pathways involved in SR resistance. Ultimately, the article presents insights into several critical areas that warrant further investigation in the study of SR-resistant mechanisms and crop breeding.

## Introduction

In the past decade, global warming has exacerbated the frequency of extreme weather events, such as high temperatures, floods and droughts, which have significantly raised the risk of crop diseases (Challinor et al. 2014). Among the more than 60 reported common diseases of agricultural crops, stalk rot (SR) is increasingly prevalent as threats to major crop production around the world in maize, wheat and rice, which is a general term of typical soil-borne diseases including stalk base rot, crown rot, soft rot and sheath rot (Ghosh et al. 2020; Guo et al. 2019; Pu et al. 2025). With the vigorous promotion of straw retention to the field and high-density planting practices, the incidence and intensity of SR disease have shown a significant upward trend (Ninkuu et al. 2025). SR, on one hand, causes lodging and directly affects the efficiency of mechanized harvesting; on the other hand, it leads to premature leaf senescence, inhibiting photosynthesis and resulting in severe yield reductions (Wang et al. 2022b). Taking the northeastern maize-producing region of China as an example, the disease incidence rate during normal years ranges from 10 to 20% (Zhang et al. 2025a). However, under extreme weather conditions, this rate can surge to between 50 and 60%, which might result in a 30% reduction in seed production (Li 2010; Khokhar et al. 2014). In wheat, crown rot can cause yield losses of up to 35% under natural inoculum levels (Kazan and Gardiner 2018; Pu et al. 2025). The disease has increasingly posed a significant threat to the Huang-Huai wheat-growing region, the foremost area for winter wheat cultivation in China (Zhou et al. 2019). The yield reduction in rice caused by SR ranges from 15 to 53% (Liu et al. 2013; Ghosh et al. 2020). In addition, the pathogens caused accumulations of a significant amount of toxins in the infected stalk and grains, posing a potential threat to the safety of livestock feed (Zhang et al. 2025a). Therefore, cultivating germplasm resources resistant to SR is of great importance for ensuring crop yield and security.

Based on the different initial infection sources, SR disease can be classified into fungal stalk rot (FSR) and bacterial stalk rot (BSR). FSR is the primary pathogenic group, involving *Fusarium*, *Pythium* and *Gibberella* (Bigirimana et al. 2015; Niu et al. 2020). FSR primarily affects the root system and the base of stalk in plants, impairing their ability to absorb nutrients effectively and reducing their resistance to lodging (Cheng et al. 2013). BSR is usually caused by Gram-negative bacteria, with representative genera including *Dickeya*, *Pseudomonas* and *Pantoea* (Chen et al. 2021; Liu et al. 2013). BSR begins with brown lesions on the leaf sheaths and root system of plant, which expand into larger patches and microtubule organisms (Zheng et al. 2006). The occurrence of crop SR disease exhibits significant regional characteristics, with the types of pathogenic bacteria closely related to temperature and humidity conditions. Low humidity conditions are conducive to the occurrence of FSR, while high temperatures combined with elevated humidity levels are more favorable for the outbreak of BSR (Chen et al. 2021). Additionally, there are some areas where both types of pathogens are present simultaneously (Niu et al. 2020). Therefore, it is essential to strengthen comparative research on FSR and BSR, which will facilitate the identification of genes and crop varieties with broad-spectrum resistance.

Despite the increasing focus on research related to crop FSR disease, there is still a lack of comprehensive and comparative reviews that address the genetic and molecular regulations underlying plant resistance to FSR and BSR diseases (Ghosh et al. 2020; Chen et al. 2021; Pu et al. 2025; Zhang et al. 2025a). Compared to several existing reviews, this review focused on the genetic and molecular regulations of resistance to SR, including key resistance loci and recent functional studies of cloned genes in maize, wheat and rice, and highlighted the application of several smart technologies to expedite the discovery of candidate genes for SR. It also systematically analyzed and discussed the similarities and differences between FSR and BSR in pathogenic mechanisms and regulation pathway of disease resistance. Finally, this review provides several insights into several critical research directions within the field of functional study and breeding strategy for resistance to SR.

## Invasive mechanisms of SR pathogens to plants

**Fig. 1:**
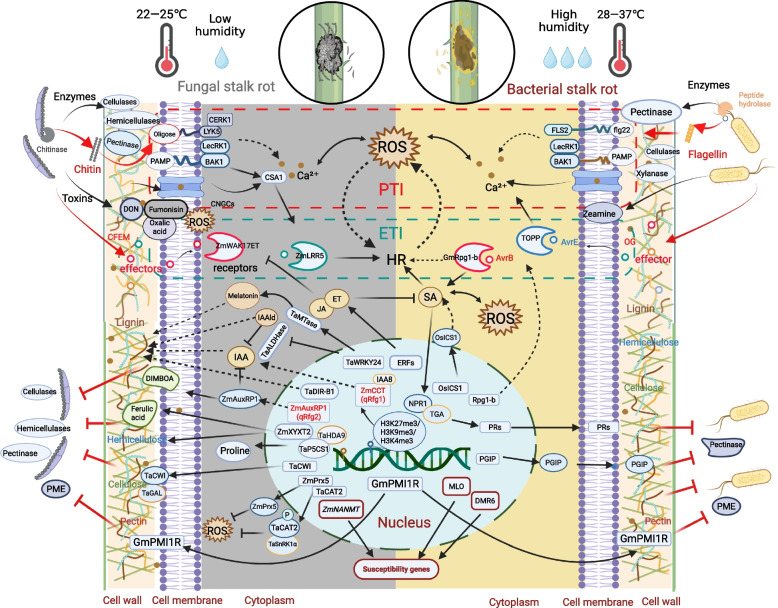
Molecular mechanisms of plant resistance to fungal and bacteria stalk rot. During the stage of pathogenic invasion of plant cells, fungi are capable of secreting a variety of cell wall-degrading enzymes and toxic factors. These enzymes, including cellulases, hemicellulases, and pectinases, primarily function to degrade the components of plant cell walls. Toxins such as fumonisins, deoxynivalenol (DON), and oxalic acid can disrupt the integrity of the cell membrane and generate reactive oxygen species (ROS). In contrast to fungi, pectinase is the primary enzyme involved in the pathogenic processes of bacteria. Once pathogens successfully disrupt the cell wall, a series of complex immune mechanisms in plants are activated, including pattern-triggered immunity (PTI) and effector-triggered immunity (ETI). For fungi, chitin in the cell wall is degraded into chitin oligomers by chitinase secreted by the plants. These chitin oligomers are recognized by the chitin receptor complex, such as LYK5 and CERK1, thereby triggering PTI. In comparison, plants secrete peptide hydrolases that degrade bacterial flagellin proteins into short peptides, such as flg22, which can be recognized by pattern recognition receptors (PRRs) like FLS2. This recognition subsequently triggers an influx of calcium ions and a burst of ROS. The complex formed by LecRK1 and BAK1 is capable of recognizing PAMPs from both bacteria and fungi. However, pathogens can secrete a multitude of effectors into host cells to evade PTI. For instance, the effector CFEM (cysteine-rich common in fungal extracellular membrane) from the fungal can be recognized by the receptor ZmWAK17ET and ZmLRP5, subsequently activating the hypersensitive response (HR). The effector AvrB, which causes bacterial stalk rot, can induce ETI mediated by GmRpg1-b. The *P. syringae* effector AvrE targets type one protein phosphatases (TOPP) protein in Arabidopsis. Both PTI and ETI can trigger the expression of a suite of disease-resistance genes. For example, *TaWRKY24*, *ZmCCT*, and *TaDIR-B1* are associated with lignin synthesis, while *ZmXYXT2* is linked to hemicellulose and lignin synthesis. *TaCWI* is related to cellulose and pectin content, and PGIP along with GmPMI1R is involved in inhibiting the secretion of pectin-degrading enzymes while maintaining a high methylesterification status of pectin to strengthen the cell wall. Additionally, *ZmAuxRP1* is associated with DIMBOA synthesis, and *Tap5CS1* is linked to proline content. *ZmPrx5* and *TaCAT2* are involved in ROS scavenging. Modification of these genes could lead to enhanced cell wall structure, reduced ROS levels, and increased production of beneficial metabolites in defense against stalk rot pathogens. In addition, the editing of susceptible (S) genes also provides an effective way to improve the broad-spectrum disease resistance of crops such as *Mlo*, *DMR6* and *ZmNANMT*. This figure was created in BioRender. https://BioRender.com/jgb7x1b

The invasion of SR pathogens is primarily facilitated by their secretion of toxins and cell wall-degrading enzymes (CWDE) to plants (Fig. 1). These biochemical agents disrupt plant defenses, allowing the pathogens to establish infection and promote disease development. For instance, fumonisins, produced by *Fusarium verticillioides*, inhibit the metabolism of sphingolipids in plants, disrupting cell membrane integrity, leading to electrolyte leakage and cell death (Kamle et al. 2019). Deoxynivalenol (DON, also known as vomitoxin), produced by *Fusarium graminearum*, interferes with ribosomal function in plants, inhibiting protein synthesis and inducing the accumulation of reactive oxygen species (ROS), which exacerbates oxidative damage (Zhang et al. 2024a). Oxalic acid, derived from *Rhizoctonia solani*, lowers the pH of plant tissues, damages cell membranes, and chelates calcium ions, weakening the cell wall structure in potato stalk (Cessna et al. 2000). Additionally, fungi typically produce various CWDE when infecting host plants. These enzymes, including cellulases, hemicellulases and pectinases, primarily function to degrade the components of plant cell walls, facilitating fungal invasion and spread. Cellulases and hemicellulases degrade cellulose and hemicellulose, disrupting the structural framework of the cell wall (Gao et al. 2025). Pectinases such as polygalacturonase (PG) break down pectin in the middle lamella, allowing for cell separation (Kubicek et al. 2014). Fungi also utilize peroxidases, such as catalase, to eliminate host ROS, thereby reducing their own oxidative damage (Heller and Tudzynski 2011). Furthermore, fungi may produce other effectors that enhance their infectivity and help them evade the immune responses of host (Lo Presti et al. 2015). Therefore, the pathogenic mechanisms of fungi encompass a wide range of factors that must be considered holistically.

Compared to pathogenic fungi, the mechanisms by which pathogenic bacteria induce SR disease exhibit both similarities and distinct characteristics (Fig. 1). The main virulence factors of pathogenic bacteria include extracellular enzymes, exopolysaccharides, lipopolysaccharides, toxins, hormones, and effectors (Chen et al. 2021). Pectinase is a mixed enzyme including PG, pectin methylesterase (PME) and pectate lyases (PL) (Haile and Ayele 2022). However, other enzymes that degrade cell wall components—such as cellulases, xylanases and proteases—also contribute to the breakdown of these structures, thereby enhancing the overall effectiveness of pectinase (Hugouvieux-Cotte-Pattat 2016). Pectinase serves two crucial functions during the infection process: first, as a metabolic enzyme, it breaks down pectin into monosaccharides, providing a vital carbon source for the pathogen’s metabolism; second, as a pathogenic factor, pectinase disrupts the integrity of the middle lamella and cell wall, leading to the lysis of plant cells (Hugouvieux-Cotte-Pattat et al. 2014). For example, pectate lyases, which are a significant category of pectinases, serve as the primary factors responsible for the maceration symptoms induced by the plant-pathogenic enterobacterium, *Dickeya* and *Pectobacterium*, which are related to soft-rot disease (Czajkowski et al. 2011). The degradation products of pectin function as signaling molecules within plants (Hugouvieux-Cotte-Pattat et al. 2014). For instance, oligogalacturonates (OGs) stimulate the accumulation of ROS and pathogenesis-related proteins, utilizing signaling pathways analogous to those triggered by pathogen-associated molecular patterns (Ferrari et al*.* 2013). Furthermore, bacterial pectinase activity is closely related to temperature. At the temperature range of 28 °C to 37 °C, the pectinase activity of *D. fangzhongdai* is notably high. Particularly at 37 °C, it significantly surpasses that of other plant pathogenic bacteria within the *Dickeya* genus and might become more aggressive (Chen et al. 2020). The pathogen responsible for BSR in rice can secrete zeamine and zeamine II toxins, which are classified as polyketide amines. These toxins directly interact with cellular structures in the host, such as the plasma membrane and chloroplasts, leading to cell death (Liu et al. 2009). Further research found *ZmsA* is essential for the synthesis of zeamine in *Dickeya zeae*, which is a significant pathogenic factor contributing to rice foot rot disease (Zhou et al. 2011). Additionally, iron metabolism and indigoidine pigment play crucial roles in the infection and symptom development in bacteria (Expert and Toussaint 1985; Reverchon et al. 2002). Therefore, intensifying research on the secreted enzymes and toxins involved in the bacterial infection process will help clarify the pathogenic mechanisms of BSR, particularly under high-temperature conditions.

## Genetic study of plant resistance to SR

### Analysis of genetic traits and QTLs to SR

To mitigate the impact of SR disease on crop production, the most effective strategy is the identification of disease-resistant loci. Extensive researches have proved the resistance of plants to SR is a quantitative trait through quantitative trait locus (QTL) mapping and genome-wide association study (GWAS) (Chen et al., 2017; Guo et al. 2019). Currently, some QTL loci related to crop SR have also been reported. Guo Cheng et al. summarized resistance loci reported in maize before 2019, which are primarily distributed on chromosomes 1, 3, 4, 5, 6, and 10 (Guo et al. 2019). Notably, the loci associated with resistance to *Fusarium* SR in maize include five QTL identified (Enrico Pè et al. 1993), along with *qRfg1* (Yang et al. 2004), *qRfg2* (Yang et al. 2010), and *qRfg3* (Ma et al. 2017). Rashid et al. (Rashid et al. 2022) conducted GWAS using 396 inbred maize lines, identifying 19 SNPs associated with resistance to *Colletotrichum* SR. Based on the GWAS results concerning field phenotypes in response to simultaneous infection with *F. graminearum* and *F. verticillioides*, 18 quantitative trait nucleotides associated with resistance were identified, and 93 candidate genes linked to resistance against synergistic infection were discovered (Gui et al. 2024). Currently, 128 loci associated with *Fusarium* crown rot resistance have been identified across all 19 wheat chromosomes, encompassing 10 resistance genes (Li et al. 2024b; Pu et al. 2025).

There have been several studies concentrated on QTL mapping of BSR. Baer et al. employed Meta-QTL analysis to identify consistent QTLs and markers associated with BSR resistance in seven biparental maize populations. The regions identified on chromosomes 1, 2, 3, 5, 6, and 10 across these populations, which exhibited significant QTL colocalization, were regarded as critical QTLs for traits related to BSR resistance (Baer et al. 2022). Mienanti et al. utilized a time-series approach in GWAS to discover SNP markers linked to BSR resistance in 624 maize lines. A particular SNP marker located on chromosome 2 demonstrated a significant association with BSR resistance throughout the entire observation period (Mienanti et al. 2024). Canama and Hautea analyzed the F_2_ population derived from the cross of P8 (BSR-susceptible) and YIF62 (BSR-resistant), identifying a major genomic region on chromosome 2 associated with resistance to BSR (*Pectobacterium chrysanthemi pv. zeae Burk., McFad.* and *Dim.*) in Tropical White Maize (Canama and Hautea 2011). Mizobuchi et al. summarized 14 QTLs pertinent to resistance against rice bacterial seedling rot and bacterial grain rot (Mizobuchi et al. 2016). Zhang et al. investigated 119 cucumber core germplasm and identified five candidate QTLs for bacterial soft rot resistance in cucumber seedlings, namely *gBSR2.1*, *gBSR2.2*, *gBSR3.1*, *gBSR4.1* and *gBSR5.1* (Zhang et al*.* 2025c). The fine mapping of these QTLs is instrumental in uncovering candidate genes for BSR resistance.

### Genetic study of plant resistance to FSR

With the increase of planting density and the promotion of straw returning measures, the FSR of crops has become more and more serious in recent years, which has brought serious challenges to the yield of crops. In order to solve these problems, a large number of genes in resistance to FSR have been carried out in maize, wheat, rice and other crops.

#### Cloning and functional characterization of key genes associated with resistance to FSR in maize

In maize, progress has been made in the cloning and identification of resistance genes against SR (Fig. 1 and Table 1). Wang et al. successfully cloned the major effect locus *qRfg1* (a CCT domain-containing gene, *ZmCCT*) resistance to *Gibberella* SR and identified a transposon-directed epigenetic change in *ZmCCT* underlies quantitative resistance (Wang et al. 2017). Upon pathogen infection, *ZmCCT* alleles without CACTA transposable element TE1 insertion can be rapidly induced and confers resistance with a transient reduction in H3K27me3/H3K9me3 (histone modifications related to gene silencing) and a gradual decrease in H3K4me3 (a histone mark associated with gene activation) in the promoter region. In contrast, the presence of TE1 insertion results in selective loss of H3K4me3 and enrichment of methylated GC, thereby suppressing *ZmCCT* expression and resulting in susceptibility to the pathogen (Wang et al. 2017). This chromatin-based regulatory mechanism allows *ZmCCT* to respond with greater precision and timeliness in defending against *F. graminearum* infection. In addition, Yang et al. proved the CACTA-like transposable element within the *ZmCCT* promoter could dramatically reduce maize flowering time (Yang et al. 2013). Ku et al. also identified the *ZmCCT* locus associated with *qRfg1* through the mapping of photoperiod-sensitive genes. These results indicate that flowering time and biological resistance are likely inherently linked, as delayed flowering is often associated with enhanced disease resistance. What is more, transcriptomic analysis of near-isogenic lines containing *ZmCCT* revealed that differentially expressed genes in these lines were enriched for genes associated with drought resistance andheat tolerance (Ku et al. 2016). ZmCCT has been shown to regulate drought tolerance by interacting with ZmFra a 1, E3 ligase ZmWIPF2 and ZmAux/IAA8, and may function as a negative regulator of ABA signaling in maize under drought stress (Zhang et al. 2024b). These findings indicate that the *ZmCCT* gene exhibits a complex functionality by involving epigenetic regulation and multiple hormone signal transduction, and may simultaneously regulate various traits and stress responses. However, the specific mechanisms by which *ZmCCT* contributes to disease resistance still need further investigation. The subsequent step involves screening and analysis for interacting proteins and downstream regulatory genes of transcription factor *ZmCCT* to validate their respective functions. Furthermore, screening excellent haplotype materials of *ZmCCT* through genetic population provides the possibility for breeding varieties with balanced growth and disease resistance.

**Table 1:** The cloned genes and their functions in regulating resistance to plant stalk rot by traditional strategies

Plant species	Gene/Protein	Gene function	Types of pathogens	Methods of genetic identification	References
Maize	ZmCCT (qRfg1)	Positive regulation of *Gibberella* stalk rot resistance	Fungal stalk rot	Map-based cloning	Wang et al. 2017
Maize	ZmAuxRP1 (qRfg2)	Positive regulation of *Gibberella* stalk rot and *Fusarium* ear rotresistance by suppressing IAA biosynthesis and promoting benzoxazinoid formation	Fungal stalk rot	Map-based cloning	Ye et al. 2019
Maize	Zm00001d051313	Positive regulation of *P. aristosporum* resistance by alter H_2_O_2_ level	Fungal stalk rot	GWAS and transcriptomics	Hou et al. 2023; Zhang et al. 2023
Maize	ZmSBR1	Positive regulation of *Fusarium* stalk rot resistance at both seedling and adult stage	Fungal stalk rot	GWAS	Song et al. 2024
Maize	ZmLecRK1	Positive regulation of both fungal and bacterial stalk rot resistance by forming immune complex with ZmBAK1 to activate broad-spectrum defense	Fungal stalk rot	GWAS	Li et al. 2024
Maize	ZmXYXT2	Positive regulation of *Fusarium verticillioides* resistance by modifying hemicellulose and lignin to strengthen cell wall structure against fungal invasion	Fungal stalk rot	Transgenic overexpression and mutant	Xu et al. 2025
Maize	ZmPrx5	Positive regulation of *Fusarium graminearum* stalk rot resistance by scavenging ROS and reprogramming defense transcriptome	Fungal stalk rot	Comparative proteomics	Wang et al*.* 2022a
Wheat	TaDIR-B1	Negative regulation of *Fusarium* crown rot resistance by affecting lignin synthesis	Fungal stalk rot	GWAS and QTL mapping	Yang et al. 2021
Wheat	TaCWI	Positive regulation of *Fusarium* crown rot resistance by increasing cellulose and pectin content through interacting with TaGAL	Fungal stalk rot	BSA and genome resequencing	Lv et al. 2023
Wheat	TaWRKY24	Positive regulation of Fusarium crown rot resistance by activating melatonin synthesis and repressing auxin production	Fungal stalk rot	Correlation analysis, transcriptome and metabolome	Xu et al. 2024
Wheat	TaMTase	Positive regulation of Fusarium crown rot resistance by catalyzing rate-limiting step in melatonin synthesis	Fungal stalk rot	Correlation analysis, transcriptome and metabolome	Xu et al. 2024
Wheat	TaCAT2	Positive regulation of *Fusarium* crown rot resistance by enhancing ROS scavenging via phosphorylation by TaSnRK1α	Fungal stalk rot	GWAS and transcriptome	Yang et al*.* 2025b
Wheat	TaALDHase	Negative regulation of *Fusarium* crown rot resistance by catalyzing IAAld to produce IAA	Fungal stalk rot	Correlation analysis, transcriptome and metabolome	Xu et al. 2024
Rice	LOC_Os09g23084	Positive regulation of sheath rot disease resistance by acting as an endoglucanase	Fungal stalk rot	Transgenic overexpression	Wan et al. 2025
Strawberry	FLS2	Receptor of flg22 and triggers an influx of calcium ions and a burst of ROS	Bacterial stalk rot	Mutant study	Weralupitiya et al. 2024
Soybean	Rpg1-b	Receptor of AvrB and activate the salicylic acid (SA) signaling pathway	Bacterial stalk rot	Effector-triggered immunity study	Selote et al. 2010
Arabidopsis	AtDMR6, AtDLO1	Negative regulation of salicylic acid and confer resistance to various classes of pathogens	Broad-spectrum resistance	Mutants	Zeilmaker et al*.* 2015
Tomato	SlDMR6-1	Negative regulation of salicylic acid and confers resistance to various classes of pathogens	Broad-spectrum resistance	Mutants	Thomazella et al*.* 2021
Maize	ZmNANMT	Negative regulation of the accumulation of nicotinate and confers resistance to various classes of pathogens	Broad-spectrum resistance	Mutants	Li et al. 2023

Additionally, Ye et al. used map-based cloning method to demonstrate that *ZmAuxRP1* is the disease resistance gene located at the *qRfg2* locus (Ye et al. 2019). *ZmAuxRP1*, which encodes an auxin-related protein localized in the plastid stroma, responded quickly to pathogen challenge with a rapid yet transient reduction in expression that led to arrested root growth but enhanced resistance to *Gibberella* SR and *Fusarium* ear rot. A model for the balance of auxin and benzoxazinoid biosynthesis mediated by *ZmAuxRP1* was proposed: under normal conditions, *ZmAuxRP1* could promote the biosynthesis of indole-3-acetic acid (IAA), while suppressing the formation of benzoxazinoid defense compounds; however, during pathogen infection, the downregulation of *ZmAuxRP1* expression redirects resources toward the production of benzoxazinoids to enhance defense mechanisms, but not the biosynthesis of IAA for growth. Thus, *ZmAuxRP1* plays a crucial role in the coordinated interaction between IAA and benzoxazinoids, effectively regulating the growth-defense balance in a timely and efficient manner to enhance plant fitness. Although this study has elucidated that *ZmAuxRP1* is integral to disease resistance through its regulatory role in the synthesis of benzoxazinoids, the potential interaction of an effector protein with *ZmAuxRP1* within the pathogen warrants further investigation. Furthermore, the potential synergistic effect of *ZmAuxRP1* in conjunction with other disease resistance genes remains to be thoroughly examined. Conducting screenings for proteins that interact with both pathogens and host plants will be crucial in assessing the existence of this mechanism. By conducting a GWAS on 295 inbred lines, Hou et al. identified 39 SNPs associated with resistance to *Pythium aristosporum* SR, which includes 69 potential resistance genes. Among these, *Zm00001d051313*, a leucine-rich repeat (LRR) family protein and receptor-like protein kinase, plays a role in maize's defense response to *Fusarium* infection (Hou et al. 2023). The transient silencing of *Zm00001d051313* facilitated *P. aristosporum* infection, thereby indicating a positive regulatory role of this gene in the antifungal defense mechanism of maize. Zhang performed a proteomic and transcriptomic analyses and identified potential roles of defence-related genes *maize chitinase 1* (*ZmCTA1), a wound- induced Bowman-Birk inhibitor (ZmWIP1), and lipoxygenase 2 (ZmLOX2)* following infection with *Fusarium verticillioides* (Zhang et al. 2023). Song et al. utilized 219 inbred lines for GWAS analysis on seedling blight resistance caused by *Fusarium verticillioides*, identifying the candidate gene *seedling blight resistance 1* (*ZmSBR1)* by both methods, which is significantly associated with resistance to *Fusarium* during the seedling stage. Phenotypic analysis of mutant lines indicated that *ZmSBR1* also confers susceptibility to SR caused by *Fusarium* in the adult stage. Haplotype 1 (CCTGA) of *ZmSBR1* not only imparts resistance to seedling blight and SR induced by *F. verticillioides* but also offers protection against SR caused by *F. graminearum* at the adult stage (Song et al. 2024). Li et al. identified the broad-spectrum resistance gene *ZmLecRK1*, a G-type lectin receptor kinase, which contributes to maize's resistance against *Fusarium* SR, northern corn leaf blight, and minor leaf spot disease, through a genome-wide association study. The resistance variant A404S of ZmLecRK1 enhances its binding affinity with the co-receptor BRI1-Associated Receptor Kinase 1 (ZmBAK1), thereby triggering downstream immune responses related to the pathways in cell wall organization or biogenesis (Li et al. 2024). The identification of these genes further substantiates that resistance to SR is a quantitative trait involving multiple loci, while GWAS provides an efficient approach for uncovering additional genes. Whether these anti-fungal SR genes with broad-spectrum resistance also play a role in BSR needs furtherverification.

#### Cloning and functional studies of important genes for resistance to FSR in wheat

Significant progress has also been made in the research on wheat SR (Fig. 1 and Table 1) (Pu et al. 2025; Ma et al*.* 2025). The majority of wheat SR is FSR, while BSR is rarely found. Lv et al. successfully cloned and identified the dual resistance gene *TaCWI*, which confers resistance to bothSBR and northern corn leaf blight. The protein encoded by TaCWI, a cell wall transferase, interacts with α-galactosidase (TaGAL), inhibiting the expression of TaGAL and its degrading effects on the cell wall. This interaction promotes cell wall thickening by increasing cellulose and pectin content, thereby enhancing resistance (Lv et al. 2023). Additionally, Yang et al. employed GWAS, nested population exon capture, and pooled transcriptomics to identify the resistance gene *catalase antioxidant enzyme 2* (*TaCAT2)* against wheat SBR. Silencing the *TaCAT2* gene resulted in a significant decrease in resistance to SBR during both seedling and adult stages, while overexpressing the *TaCAT2* gene significantly enhanced resistance at both growth stages. The interaction between TaCAT2 and a sucrose non-fermenting-1-related protein kinase alpha subunit (TaSnRK1α)was validated and the phosphorylation of TaCAT2-R by TaSnRK1α enhances its protein stability, thereby improving its ability to scavenge ROS within the plant and modulating resistance to wheat SBR (Yang et al. 2025b). Through associative analysis, they identified that *TaWRKY24* can activate the rate-limiting enzyme gene for melatonin synthesis, TaMTase, and maintain its activity to enhance disease resistance. Simultaneously, *TaWRKY24* inhibits the auxin synthase gene *TaALDHase*, leading to reduced levels of IAA and increased levels of IAAld, thereby further conferring resistance to diseases (Xu et al. 2024). Moreover, Yang et al. identified a dirigent protein candidate gene *TaDIR-B1* that regulates resistance to wheat SBR by GWAS of 435 wheat introgression lines. The functional loss of this gene significantly enhances the resistance of wheat, potentially through the increased accumulation of lignin content to harden plant cell wall structure (Yang et al. 2021). However, the mechanism of how *TaDIR-B1* participates in lignin synthesis remains to be further studied. The evidence might be provided by studying the interaction between *TaDIR-B1* and laccase. These results indicate that genes associated with cell wall structure and ROS scavenging play a significant role in wheat's resistance to SR.

#### Cloning and functional studies of important genes for resistance to FSR in rice

In comparison to maize and wheat, research on the associated genes in rice regarding sheath rot is still limited (Table 1). SR of rice, caused by *Sclerotium or Sarocladium oryzae*, has emerged as a significant fungal disease, responsible for approximately 35% of crop losses. The activation of MAPKs, regulation of the *npr1* gene, abundant expression of transcription factor genes, and the release of antimicrobial PR proteins may be harnessed through biotechnological tools to enhance stalk rot resistance in rice (Ghosh et al. 2020). The activity of phenylalanine ammonia lyase (PAL), catalase (CAT), superoxide dismutase (SOD), and total phenols were much higher in resistant genotypes compared to susceptible ones. Furthermore, the expression levels of pathogenesis-related genes, including *PR-1a*, chitinase (*Cht-1*), and beta-1,3-glucanase (*Gns1*), were markedly elevated in resistant genotypes (Behura et al. 2025). Wan et al. found overexpression of *LOC_Os09g23084*, encoding an endoglucanase-1 precursor, resulted in a decrease in rice development and an increase in susceptibility to sheath rot disease at the harvest stage (Wan et al. 2025). Nevertheless, the impact of *LOC_Os09g23084* on plant growth, as well as its influence on cell wall composition and structure, remains ambiguous. Thus, it is imperative to investigate whether such genes, which encodes an endo-(1,4)-β-glucanase, are conserved across different species and to assess the disease resistance conferred by various genotypes from a population genetics perspective. This approach will aid in enhancing the resistance of crops to stalk rot.

### Genetic study of plant resistance to BSR

By employing GWAS and QTL mapping strategies, numerous candidate genes related to BSR have been identified and functionally characterized. For instance, *Defective Kernel 1* (*DEK1*) has been mapped and shown to have a strong correlation with BSR (Baer et al. 2022). *DEK1* has been reported to be associated with a mechanically activated Ca^2+^ current in plants (Tran et al. 2017). This suggests that mechanical stress induced by BSR infection may affect maize plants. *Zm00001eb093950*, which encodes a Calcium-dependent protein kinase (CDPK), presumably plays a crucial role in mediating BSR resistance in maize by facilitating the plant's response to biotic stresses (Hufford et al. 2021). Additionally, *Zm00001eb033030*, which encodes DIMBOA UDP-glucosyltransferase BX9, may serve as a significant early defense mechanism against a wide range of pathogens, including BSR (Mienanti et al. 2024). Six candidate genes, including *CsaV3_2G014450*, *CsaV3_2G014490*, *CsaV3_2G016000*, *CsaV3_3G000850*, *CsaV3_4G033150*, and *CsaV3_5G000390*, were significantly upregulated in the resistant genotypes following inoculation for bacterial soft rot resistance in cucumber seedlings (Zhang et al*.* 2025). Despite the identification of these genes associated with BSR, their specific biological functions still necessitate genetic validation through the development of overexpression and gene editing materials.

### Genetic study of susceptible genes for broad-spectrum resistance to SR

Disabling susceptibility (S) genes presents a promising alternative to R genes in breeding programs, as it typically provides durable and broad-spectrum disease resistance, including against BSR. For instance, *downy mildew resistant 6* (*DMR6*) and *DMR6-LIKE OXYGENASEs* (*DLOs*), both of which belong to the superfamily of 2-oxoglutarate Fe (II)-dependent oxygenases, encode enzymes in Arabidopsis and its orthologs in other plants such as tomato and banana, identified as susceptibility factors to bacterial and oomycete pathogens (Zeilmaker et al*.* 2015). The inactivation of *AtDMR6* leads to increased levels of salicylic acid (SA; 2-hydroxybenzoic acid) and confers resistance to various classes of pathogens, including the bacterium *Pseudomonas syringae* and the oomycete *Phytophthora capsici* (Zeilmaker et al*.* 2015). Moreover, the *dmr6-3dlo1* double mutant exhibits complete resistance to *H. arabidopsidis*; however, this is accompanied by significant growth reduction correlated with elevated salicylic acid levels.

*SlDMR6-1* and *SlDMR6-2*, two orthologs in tomato, have undergone subfunctionalization, with *SlDMR6-2* potentially specializing in balancing SA levels during flowering and fruit development, while *SlDMR6-1* retains the capacity to fine-tune SA levels during pathogen infection (Thomazella et al*.* 2021). Notably, *SlDMR6-1*, but not *SlDMR6-2*, is upregulated in response to pathogen infection. *Sldmr6-1* mutants demonstrate enhanced resistance against various pathogens, including bacteria, oomycetes, and fungi. In contrast, *SlDMR6-2* does not contribute to immunity in vegetative tissues and is primarily restricted to reproductive organs, such as flowers and immature fruits. This research not only reinforces the mechanism underpinning SA homeostasis in plants but also offers a promising strategy for engineering broad-spectrum and durable disease resistance in crops (Thomazella et al*.* 2021). Future investigations on *DMR6* will involve identifying mutations in the promoter regulatory elements that downregulate *SlDMR6-1*, with the objective of sustaining heightened pathogen resistance while minimizing adverse effects on plant growth and development in the field. Furthermore, CRISPR/Cas9-mediated editing of the *DMR6* ortholog in banana (*Musa spp.*) has been shown to confer enhanced resistance to bacterial *Xanthomonas wilt* disease caused by *Xanthomonas campestris pv. musacearum* (Xcm) (Tripathi et al. 2021). Thus, gene editing of the *DMR6* and *DLO* orthologs in major crops could provide valuable targets for improving bacterial resistance and achieving broad-spectrum resistance.

*ZmNANMT*, *nicotinate N-methyltransferase 1*, was identified as a novel plant susceptibility gene and genome editing of this gene causes a quantitative effect on increasing multiple disease resistance in maize without agronomic penalty by increasing the accumulation of nicotinate (Li et al*.* 2023). Compared with the influence scope of FSR disease, the influence scope of BSR disease is still less, but with the change of global climate, the occurrence of BSR disease shows an increasing trend. Although some studies have been carried out in plants, the molecular regulatory network of plant response to bacterial diseases needs to be further strengthened.

### Leveraging cutting-edge strategies to expedite the discovery of candidate genes for SR

Single-cell sequencing technology (SC-RNA-seq) has recently emerged as a powerful and smart technology for elucidating cellular heterogeneity by overcoming the averaging limitations of traditional bulk sequencing methods. Cao et al. utilized SC-RNA-seq to construct a high-resolution cellular atlas of maize root tips, and discovered that the maize root apical meristem possesses innate immunity against the SR pathogen *Fusarium verticillioides*. Differentially expressed genes *ZmWOX5b* and *ZmPIN1a* regulate the biosynthesis of indole IAA, facilitating the formation of a critical IAA concentration gradient in the root tips, thereby promoting root elongation to protect the RAM. Additionally, the research validated that genes involved in the phenylpropanoid metabolic pathway (*ZmPAL6*, *ZmCOMT*, and *ZmCCoAOMT2*) participate in cell type-specific disease defense by regulating lignin synthesis (Cao et al. 2023). In the future, by leveraging SC-RNA-seq, we will investigate how different tissues and cells in maize stalks, ears, and leaves respond to pathogen invasion, which will significantly enhance our ability to uncover additional disease resistance genes.

The application of artificial intelligence (AI) technology has provided another powerful strategy for the targeted design of specific disease-resistant proteins. For instance, GmPMI1, a plant-derived pectin methylesterase inhibitor protein, safeguards pectin by maintaining a high level of methylesterification, thereby resisting pathogen invasion. However, the constitutive expression of GmPMI1 disrupts the balance between host growth and defense responses. To address this issue, a modified variant of GmPMI1, referred to as GmPMI1R, was designed using AlphaFold2-Multimer software. Nine residues of GmPMI1 were selected for mutation, which markedly reduced the formation of hydrogen bonds with GmPME1, while not significantly impacting its interaction with PsPME1. This modification significantly enhances the broad-spectrum resistance of soybeans to oomycetes and fungi without compromising growth by specifically targeting and inhibiting microbial pectin methylesterases (Xia et al. 2024). Another example involves the engineering of polygalacturonase-inhibiting proteins (PGIPs) in *Phaseolus vulgaris* to achieve broader specificity towards *Fusarium phyllophilum* polygalacturonase (FpPG) (Xiao et al. 2024). Structure-based engineering by mutation transforms a putative PGIP that initially lacks FpPG-binding activity into a highly effective FpPG-interacting protein, facilitating the production of long-chain immunoactive oligogalacturonides. Thus, predicting and designing the sequence and structure of crucial disease resistance genes using AIalgorithms offer an effective solution to the challenge of balancing plant resistance and growth. This approach will help us find the key targets efficiently and aid in the cultivation of new crop varieties that exhibit both high yield and enhanced disease resistance.

Synthetic biology can also provide an innovative strategy in enhancing plant broad-spectrum disease resistance by remodeling plant immune receptors. Wang et al. (2025) report a single engineered NLR can confer plant broad-spectrum and complete plant resistance against multiple potyviruses (Wang et al. 2025). Through fusing a flexible blocking polypeptide containing conserved pathogen-derived protease cleavage sites (PCSs) to the N-terminus of an engineered aNLR, a "safety lock" was created at this N-terminal tag, preventing the aNLR from triggering defense prematurely. Ultimately, by weaponizing the pathogen’s cleavage machinery—an indispensable survival mechanism—as a self-destruct switch, the strategy achieves broad-spectrum, complete, and durable resistance (Wang et al. 2025; Wu et al. 2025). This strategy holds the potential to be exploited to control viruses, bacteria, oomycetes, fungi, nematodes and pests affecting plants. In addition, the interfamily co-transfer of sensor and helper NLRs can enhance the applicability of sensor NLRs, thereby expanding the array of tools available for the management of diseases in rice, soybean, Brassica, and other crops (Du et al. 2025). By introducing the sensor NLR (Bs2), which recognizes bacterial effectors in chili peppers, alongside the helper NRC-type receptors from tobacco into rice, the transgenic rice can induce the oligomerization of NRC in vivo after inoculation with *Xoc*. This also triggers the expression of defense-related genes, indicating that the synergistic expression of both receptors successfully reconstructs the immune pathway in rice (Du et al. 2025). This study systematically validates, for the first time, the synergistic strategy of "sensor + helper" NLR immune receptors in reconstructing immune pathways in distantly related crops. This serves as an excellent example for the application of biological breeding utilizing NLRs from other species, particularly in light of the absence of endogenous disease resistance genes within germplasm resources.

To systematically analyze complex biological phenomena, the establishment of an integrative multi-omics database incorporating genomic, transcriptomic, degradomic, proteomic, and metabolomic data across multiple layers is essential. Zhang et al. constructed the first high-throughput multi-omics atlas of wheat, encompassing transcriptomic, proteomic, phosphoproteomic, and acetylomic data from 20 different tissues throughout the entire growth cycle. This comprehensive atlas led to the discovery of a unique protein module, TaHDA9-TaP5CS1: upon infection by the pathogen *Fusarium pseudograminearum* (F. pg), the expression of *Histone Deacetylase 9* (*TaHDA9)* decreases in resistant plants, relieving its acetylation repression of *TaP5CS1* (a potential Δ1-pyrroline-5-carboxylate synthetase) and promoting the accumulation of proline, thereby significantly enhancing resistance to basal SR (Zhang et al. 2025b). Through a comparative proteomics approach at identifying candidate proteins associated with stalk rot resistance against *Fusarium graminearum*, Wang et al. discovered ZmPrx5, an antioxidant enzyme that scavenges ROS, positively regulates resistance against stalk rot in maize, likely through defense-oriented transcriptome reprogramming (Wang et al. 2022a). Through the analysis of sRNA, degradome, and transcriptome sequencing results in response to SR, the module of zma-miRNA319-ZmMYB74 was identified as a regulator of maize resistance to SR by modulating lignin deposition (Cao et al. 2025). ZmMYB74 negatively regulated the expression of *Cinnamyl alcohol dehydrogenase* (*ZmCAD*), resulting in diminished lignin biosynthesis and weakened resistance to SR. Conversely, the knockout or suppression of *ZmMYB74* markedly enhanced resistance to SR. Therefore, multi-omic data can provide a hologram for the mining and functional analysis of SR resistance genes.

## Molecular regulation pathwaysof plant resistance to SR

Although a certain number of candidate genes associated with crop resistance to SR have been identified through forward and reverse genetics, as well as through innovative approaches (Tables 1 and 2), the gene functions and pathways implicated in disease resistance have yet to be fully elucidated. Since the trait of plant disease resistance to SR has been proved to be a complex quantitative that might involve multiple regulatory pathways like other plant disease (Deng et al. 2020; Gou et al*.* 2023; Wan et al. 2021; Wang et al. 2022a), we systematically summarized the possible regulation pathways involved in plant resistance to SR by referring to the published disease resistance literature (Fig. 1).

**Table 2:** The cloned genes and their functions in regulating resistance to plant stalk rot by novel strategies

Novel strategies	Applications	Key findings	References
Single-cell sequencing technology	Construct a high-resolution cellular atlas of maize root tips conditioning fungal invasion and identify disease genes	Identify six cell type-specific immune regulatory networks, 16 known maize disease-resistant genes, five experimentally validated genes, 42QTL predicted genes; *ZmWOX5b* and *ZmPIN1a* positively regulate *Fusarium verticillioides* resistance by maintaining root apical meristem immunity via IAA gradient regulation	Cao et al. 2023
Artificial intelligence technology	Achieve targeted design of specific disease-resistant proteins	GmPMI1R, markedly reduced the formation of hydrogen bonds with GmPME1, while not significantly impacting its interaction with PsPME1; Mutated PGIP enhanced the interaction with FpPG and facilitated the production of long-chain immunoactive oligogalacturonides	Xia et al. 2024; Xiao et al. 2024
Synthetic biology	Enhance plant broad-spectrum disease resistance by remodelling plant immune receptors	A single engineered NLR can confer broad-spectrum and complete resistance against multiple potyviruses; "sensor + helper" NLR immune receptors in reconstructing immune pathways in distantly related crops	Wang et al. 2025; Du et al. 2025
Multi-omics database	Systematically analyze complex biological phenomena against SR	A unique protein module, TaHDA9-TaP5CS1; zma-miRNA319-ZmMYB74 module regulate maize resistance to SR	Zhang et al. 2025b; Cao et al. 2025

### Plant cell wall-mediated disease resistance pathway

To combat pathogen infections, plants have developed multi-tiered passive and active resistance mechanisms throughout the course of evolution (Jian et al. 2023). The cell wall initially acts as a passive barrier that pathogens must degrade by secreting CWDE to facilitate the progression of infection (Molina et al. 2021). In addition, previous studies have identified the intermediate compounds derived from cell wall components may function as antimicrobial agents or phytoalexins against pathogens (Barber et al. 2000). Furthermore, alterations in the cell wall may also initiate disease resistance responses (Bacete et al. 2018). Therefore, the components and structural characteristics of cell wall significantly influence the disease resistance of plants (Fig. 1).

Plants construct this barrier to resist pathogens by dynamically modifying components such as lignin, hemicellulose, pectin and cellulose (Jaafar and Anderson 2024; Munzert and Engelsdorf 2025). The composition of lignin could influence the release of latent defense signaling molecules from the cell wall, thereby impacting elicitor-activated defense responses (Gallego‐Giraldo et al. 2018). For example, *ZmMYB31* likely inhibited resistance to SR by reducing lignin content (Xie et al. 2025). The *zmmyb31* variant exhibited greater resistance than the wild-type and possessed a higher lignin content. Hemicellulose is another significant component of the plant cell wall which consists of a variety of polysaccharides (Scheller and Ulvskov 2010). For example, the overexpression of *Xylan Xylosyltransferase 2* (*ZmXYXT2*) induces significant modifications in the composition of maize cell walls, notably elevating the levels of arabinose ande xylose. These changes lead to cell wall thickening, which effectively hinders the intracellular invasion and colonization by *F. verticillioides*, thereby preventing the spread of the pathogen between cells (Xu et al. 2025). Pectin metabolism has been proved to play a crucial role in cell-wall integrity, detection of plant pathogens, and defense response (Wang et al. 2023a). Cellulose comprises approximately one-third of the cell wall, thereby serving as a crucial component of the plant cell wall and being recognized as the most abundant biological polymer on earth (Zhang et al. 2021). However, the role of cellulose synthesis in resistance to SR disease requires further research (Molina et al. 2024).

The cell wall also serves as a participant in stress signal transduction through cell wall integrity receptors (Wang et al. 2023b). During the infection process, pathogens secrete various CWDE, including cellulases, pectinases, and xylanases, which degrade the plant host's cell wall and generate certain oligosaccharide elicitors. These oligosaccharide elicitors can act as damage-associated molecular patterns (DAMPs), triggering different signaling pathways to regulate the plant's immune response and further enhance disease resistance (Wan et al. 2021). Therefore, the dynamic remodeling of plant cell walls might play a crucial role in response to both fungi and bacteria SR, which warrant further investigation.

### The biosynthesis of plant secondary metabolites in resistance to SR

Another type of passive defense mechanism in plants is the biosynthesis of toxic metabolites against pathogens. Examples of secondary metabolites encompass various chemicals that are frequently employed as antimicrobial agents in plants, including phenylpropanoids, benzoxazinoids, saponins and glucosinolates (Piasecka et al. 2015). For example, the ferulic acid (FA) content in the stalks of disease-resistant wheat varieties is markedly higher than that in susceptible varieties. FA could induce the disruption of cell surface morphology and target cell membrane (Yan et al. 2023). Overexpression of *ZmXYXT2* could elevate the levels of FA and increase the resistance to *F. verticillioides* (Xu et al. 2025). Using comprehensive targeted metabolomics combined with transcriptomics in resistant and susceptible wheat varieties inoculated with *Fusarium graminearum* (WZ-8A), the results revealed that both benzoxazolin-2-one (BOA) and 6-methoxy-benzoxazolin-2-one (MBOA) significantly inhibited the growth of the pathogen. A typical representative from the *Poaceae* family, 2,4-dihydroxy-1,4-benzoxazin-3-one (DIMBOA), when exogenously applied, can significantly reduce the activity of cell wall degrading enzymes produced by *Fusarium graminearum* and decrease the accumulation of toxins such asFA (Ma et al. 2024). For example, the downregulation of *ZmAuxRP1* expression reallocates resources towards the synthesis of benzoxazinoids, such as DIMBOA, thereby strengthening defense mechanisms (Ye et al. 2019). Notably, these defensive metabolites, such as phenylpropanoids, are not evenly distributed; instead, they preferentially accumulate at infection sites or during specific developmental stages. This localized expression mechanism effectively reduces energy expenditure and enhances defensive efficiency. Such precise spatial and temporal accumulation is often regulated by epigenetic modifications, such as histone acetylation, and cell type-specific promoters, which requires further research (Ferreira and Antunes 2024). Furthermore, it is essential to identify other metabolites capable of inhibiting BSR pathogens. For instance, alterations in sugar metabolism pathways within plants could influence their interactions with bacterial pathogens (Liu et al. 2022).

### Plant messenger-mediated disease resistance pathway to SR

Ca^2^⁺ is one of the most highly conserved messengers in plant immunity. As a ubiquitous signaling molecule, Ca^2^⁺ governs a broad array of cellular metabolic processes, including the regulation of oxidative bursts, gene expression, and signal transduction; it also modulates several critical stages in the apoptotic process (Aldon et al. 2018). The concentration of Ca^2^⁺ is vital for immunity triggered by Ca^2^⁺-dependent pathogen-associated molecular patterns (PAMPs) in plants (Mohanta et al. 2019; Tian et al. 2019). Recent studies have indicated that Calcium-modulated proteins (CMLs), including CML13 and CML8, play a significant role in plant defense responses against various pathogens, such as *Pseudomonas syringae*. The overexpression of these proteins has been shown to regulate pathogenesis-related genes, as well as several genes involved in signal transduction and stress responses (Zhu et al. 2016; Zhu et al. 2021). Thus, the role of calcium ions in plant response to SR cannot be ignored.

ROS are not only byproducts of regular metabolic processes, but they also play a critical role as signaling messengers in the first line of plant defense against pathogen invasion (Mittler et al. 2022). In plants, ROS are produced in many organelles including apoplasts, chloroplasts, mitochondria and peroxisomes. ROS play dual roles in plant defense responses. Although ROS become toxic when their levels surpass a certain threshold, low concentrations of ROS can function as crucial signaling molecules for plant growth and development, as well as for plant responses to abiotic and biotic stresses (Fichman and Mittler 2020). For example, CAT could regulate ROS to mediate plant resistance, and the overexpression of *TaCAT2* gene in plants resulted in increased resistance to wheat *Fusarium* crown rot (Yang et al. 2025b). Additionally, ROS are involved in the lignification of the cell wall and the cross-linking of associated proteins, thereby strengthening the cell wall and enhancing the structural disease resistance of the host (Wang et al. 2024). In addition to Ca^2^⁺ and ROS, there are other significant disease-resistant signaling molecules that need to be identified, and their interactions and cross-regulatory mechanisms require further in-depth analysis.

### Plant hormone-mediated disease resistance pathway

Phytohormonal crosstalk has been proved to play a crucial role in the biotic stresses (Ning et al. 2017). SA signaling plays a pivotal role in rice resistance to SR disease, primarily by inducing the production of pathogenesis-related proteins to enhance resistance. For example, SA proficiently suppressed the mycelial proliferation of *Rhizoctonia solani* and markedly diminished necrosis, chlorosis, and collar rot in *Capsicum chinense* (Gogoi et al. 2025). *Arabidopsis thaliana* Calcium-Dependent Protein Kinase 5 (CDPK5) enhances SA-mediated resistance to the bacterial pathogen *P. syringae pv.* tomato strain DC3000, leading to differential expression of plant defense genes and the synthesis of ROS (Dubiella et al. 2013). Although SA has some efficacy against certain fungal and bacterial diseases, its action is often antagonistically suppressed by jasmonic acid (JA) and ethylene (ET) signaling pathways (Landi et al. 2025).

In contrast, JA and ethylene ET signaling pathways typically work synergistically to regulate plant defenses against fungal pathogens (Trang Nguyen et al. 2019; Adie et al. 2007). This synergistic interaction primarily occurs through the integration of signals via interactions among transcription factors, collectively activating downstream defense genes such as lipoxygenase (*LOX*), plant defensin gene (*PDF1.2*), and ethylene response factors (such as *ORA59*) (Haghpanah et al. 2024). Furthermore, it has been discovered that auxin signaling and SA signaling exhibit antagonistic interactions during the process of lateral root development, collectively participating in the regulation of the mechanisms through which pathogens invade via lateral roots (Kong et al. 2020). However, the mechanisms by which hormonal balance promotes growth and enhances disease resistance to FSR and BSR require further elucidation.

### Plant immune systems against SR

#### PAMP-Triggered immunity

When pathogens successfully infect plants, the plants activate a series of complex immune mechanisms to prevent the invasion (Fig. 1). Plants utilize plasma membrane-localized pattern recognition receptors (PRRs) to directly recognize PAMPs, thereby initiating the first layer of their immune system—PAMP-triggered immunity (PTI) (Jones and Dang 2006). PTI is widely conserved across various plant species and serves as a fundamental line of defense against a diverse range of pathogens. For example, Fp00392 and Cell Death-induced Protein 1 (FpCDP1) were two proteins identified in recent studies as conserved PAMPs in *F. pseudograminearum* (Liu et al. 2025; Yang et al. 2025a). When wheat perceives PAMPs, the cytosolic calcium concentration exhibits a biphasic fluctuation: the first peak drives the production of ROS through NADPH oxidase, while the second peak activates transcriptional reprogramming (Bhar et al. 2023). This conserved calcium-ROS regulatory module is also present in maize and soybean, although different species may achieve signal amplification through distinct combinations of genes.

In response to various PAMPs associated with pathogens, plants possess specific PRR proteins that recognize these signals and trigger the PTI response (Wang et al. 2022a). There are certain differences between the PAMP proteins of fungi and bacteria. For example, when fungi invade plants, the plants secrete chitinase, which degrades the chitin in the fungal cell wall into oligoses. These chitin oligomers were recognized by chitin receptor complex such as LysM-containing receptor-like kinase 5 (LYK5) and Chitin Elicitor Receptor Kinase 1 (CERK1), thereby triggering PTI (Gao et al. 2019; Gong et al. 2020). In comparison, when bacterial pathogens infect plants, the plants secrete peptide hydrolases that degrade the bacterial flagellin proteins into short peptides such as flg22, subsequently triggering the plant's PTI response (Wei et al. 2020). Upon recognition of flg22 by PRRs located on the plant cell membrane, such as FLS2, an influx of calcium ions and a burst of ROS are triggered, activating the expression of downstream defense genes (Weralupitiya et al. 2024). The complex formed by ZmLecRK1 and ZmBAK1 was capable of recognizing PAMPs from both bacteria and fungi (Couto and Zipfel 2016; Li et al. 2024). Furthermore, the monosaccharides derived from the degradation of the cell wall can also function as signaling molecules, triggering the PTI response (Wan et al. 2021).

#### Effector-triggered immunity

However, pathogens can secrete a myriad of effectors into the host cells to evade PTI (Chisholm et al. 2006; Boller and He 2009; Wang et al. 2022a). These immune receptors, including intracellular nucleotide-binding domain and leucine-rich repeat (NLR) proteins, recognize microbial effectors either directly or indirectly, thereby triggering a swift and robust defensive response known as effector-triggered immunity (ETI) (Gu et al. 2025). ETI is often linked to rapid programmed cell death (PCD) and ROS burst, commonly referred to as the HR, at sites of infection. ETI has been extensively utilized in crop breeding to enhance resistance traits (Dangl et al. 2013).

Taking the FSR disease of crops as an example, related studies have discovered that cysteine-rich common in fungal extracellular membrane (CFEM) domain proteins of *Fusarium graminearum* could functionalize as an effector and interact with maize receptors like ZmWAK17ET (an alternative splicing isoform of ZmWAK17) or ZmLRR5 (Zuo et al. 2022). ZmLRR5 and ZmWAK17ET could interact with the extracellular domain of ZmWAK17 to trigger cell death. The overexpression of *ZmWAK17*shows increased resistance to *F. graminearum*, while loss-function of *ZmWAK17* exhibit enhanced susceptibility to *F. graminearum*. The co-expression of CFEMs with ZmWAK17ET or ZmLRR5 inhibits the cell death induced by ZmWAK17. This finding indicated that *ZmWAK17* plays a crucial role in mediating resistance to FSR, while *F. graminearum* introduces apoplastic CFEMs that undermine ZmWAK17-mediated resistance (Zuo et al. 2022). Additional fungal effectors can be identified by assessing their interactions with plant disease resistance genes in the future.

BSR pathogens primarily utilize a dual strategy that involves the precise disruption of immune signaling through effectors, as well as the direct damage or alteration of physiological processes (Song et al. 2022a). For example,a well-studied bacteria effector Avirulence B (AvrB) from *Pseudomonas syringae*, can induce ETI mediated by the soybean gene *Rpg1-b* through interacting with RIN4-like proteins (Selote and Kachroo 2010). Another example is the Avirulence E (AvrE) effector, which is one of the type III secreted effectors that are highly conserved among numerous agriculturally significant phytopathogenic bacteria (Herold et al. 2025). AvrE homologs have been identified in other species of *Pseudomonas*, as well as in various genera, including *Pantoea*, *Erwinia*, *Dickeya*, and *Pectobacterium*. Hu et al. discovered that the *P. syringae* effector AvrE targets type one protein phosphatases (TOPP) protein in Arabidopsis, thereby modifying abscisic acid (ABA) signaling, which is essential for the water-soaking phenomenon and disease caused by *P. syringae* (Hu et al. 2022)In contrast to *Pseudomonas*, T3SS and T3 effectors do not seem to be central to the pathogenesis of pectobacteria. Instead, oligogalacturonide (OG) fragments released by bacterial pectin-degrading enzymes may be recognized as DAMPs that activate innate immune responses (Davidsson et al. 2013). Although these effectors from *Pseudomonas* bacteria and interacted proteins within plants have been identified, whether these effector-Triggered immunity also play a role in BSR remains to be further studied. The research in other pathogenic bacteria can provide us with good reference to study BSR. These studies reveal the different mechanism that bacterial effectors tend to directly target and interfere with the immune signaling components themselves, while fungal effectors often exploit polymorphism to evade recognition by NLR proteins.

## Conclusions and prospects

The incidence of basal SR diseases is increasing globally, presenting significant challenges to crop yield, quality, and food safety. The mechanisms by which plants resist SR are complex and represent an important quantitative trait. This article systematically reviews the infection mechanisms of fungal and bacterial pathogens causing SR, the currently identified resistance QTLs and genes through traditional and modern technologies based on recent literatures, and the various physiological and molecular pathways of plant resistance to SR. Despite significant advancements in plant resistance to SR, there remain several critical areas that require in-depth and systematic investigation.

### Strengthening researches on the BSR and exploration of broad-spectrum disease resistance genes to SR

In recent years, the increasing frequency of high temperature and moisture conditions has driven the northward spread of BSR, expanding its infection range (Chen et al. 2021). Compared to FSR, the pathogenic mechanisms of BSR exhibit certain differences. For instance, pectinase is the primary enzyme secreted by bacterial pathogens during their invasion of plant cells, and the activity of pectinase is positively correlated with increasing temperatures within a certain range (Hugouvieux-Cotte-Pattat 2016; Chen et al. 2020). Bacterial flagellin proteins serve as significant PAMPs, while fungi possess chitin structures, both of which play distinct roles in triggering plant PTI (Wang et al. 2022a). However, the mechanisms by which plants specifically perceive bacterial PAMPs, recognize their effectors, and activate effective defense signaling pathways remain unclear. Future research urgently needs to identify the elite germplasms and genes in resistance to BSR, elucidate how bacteria manipulate host hormones and plant defenses, as well as to explore the specific changes in resistance mechanisms under high-temperature conditions.

Researches have indicated that fungal and bacterial pathogens can co-infect plants, exacerbating the level of damage to plant cells (Jian et al. 2023). Therefore, the interactions and cooperative patterns between fungal and bacterial pathogens require further elucidation. Furthermore, it is essential to enhance research on whether the defense mechanisms of plants against similar virulence factors are conserved in FSR and BSR. Such studies will facilitate the screening of superior germplasm resources resistant to both fungal and bacterial co-stress basal SR and the identification of broad-spectrum key genes associated with these resistances. In addition, the editing of susceptible genes also provides an effective way to improve the broad-spectrum disease resistance of crops such as *Mlo*, *DMR6* and *ZmNANMT* (Tripathi et al. 2021; Wang et al. 2014; Li et al*.* 2023).

### Deepening the understanding of the plant growth-defense balance mechanism

Plant SR is prevalent in the main crops such as maize, wheat and rice, and several important disease-resistant genes have been cloned. However, these genes, while involved in disease resistance, are also closely associated with plant growth and other traits. For instance, The *ZmCCT* gene (*qRfg1*) is not only associated with maize SR disease but also plays a role in the regulation of flowering time (Wang et al*.* 2017). The expression levels of *ZmAuxRP1* (*qRfg2*) play a crucial role in balancing the biosynthesis of auxin levels for normal growth and benzoxazinoids for disease defense (Ye et al. 2019). Given that the environmental conditions for plant growth are dynamically changing, it is essential to enhance research on the specific molecular mechanisms by which these disease-resistant genes regulate growth-defense trade-off (He et al. 2022).

### Development and integration of multi-dimensional technologies for research on plant disease resistance

With the advancement of several cutting-edge technologies and bioinformatics, the molecular networks underlying various important traits in crops have been extensively elucidated (Wu et al. 2021). The processes of plant pathogenesis and resistance are inherently complex, necessitating the utilization of multi-omics technologies to aid in the analysis of pathogen mechanisms and the exploration of plant resistance genes, particularly at the population level. These omics approaches include GWAS, QTL sequencing (QTL-seq), genomics, transcriptomics, proteomics, protein interaction networks, epigenomics, single-cell omics, gene editing, and synthetic biology based on AI-driven protein redesign. The application of these multi-dimensional methods and technologies significantly contributes to the systematic analysis of plant resistance mechanisms.

### Future breeding strategy for resistance to SR

Enhancing genetic and molecular research on FSR and BSR in crops will facilitate the future breeding of resistant varieties to SR. Firstly, the identification of disease-resistant materials and superior haplotypes through various populations, including inbred lines and wild germplasm resources, could be achieved by GWAS and QTL-mapping. This involves the development of functional molecular markers and the screening of disease-resistant inbred lines as foundational resources for disease resistance breeding (Veerendrakumar et al. 2025). Second, multi-dimensional omics technology has been employed to efficiently identify both disease-resistant and susceptible genes. Gene editing technology is utilized to modify the coding and promoter regions of these genes to produce broad-spectrum disease-resistant materials, and select elite mutations for addressing the challenge of balancing disease resistance with growth (Rodríguez-Leal et al. 2017). Furthermore, haploid breeding is implemented to accelerate the breeding process (Maruthachalam 2024).

## Data Availability

Not applicable.

## Abbreviations

ABA: Abscisic acid
BSR: Bacterial stalk rot
Ca^2^⁺: Calcium ion
CWDE: Cell wall-degrading enzymes
DAMP: Damage-associated molecular patterns
DON: Deoxynivalenol
ET: Ethylene
ETI: Effector-triggered immunity
FA: Ferulic acid
FSR: Fungal stalk rot
GWAS: Genome-wide association study
HR: Hypersensitive response
IAA: Indole-3-acetic acid
JA: Jasmonic acid
NLR: Leucine-rich repeat
OG: Oligogalacturonide
PAMP: Pathogen-associated molecular patterns
PCD: Programmed cell death
PG: Polygalacturonase
PME: Pectin methylesterase
PMI: Pectin methylesterase inhibitor
PRRs: Pattern recognition receptors
PTI: PAMP-triggered immunity
QTL: Quantitative trait locus
ROS: Reactive oxygen species
SA: Salicylic acid
SR: Stalk rot
