# Correction: Advances in genetic and molecular mechanisms of crop resistance to stalk rot

**DOI:** 10.1007/s44154-026-00322-4

**Published:** 2026-06-05

**Authors:** Yueqi Kang, Mingxiu Ruan, Qinhan Hu, Zihao Gui, Jinyan Zhou, Jianbo Yao, Yuanyuan Cao, Ting Ding, Bo Wang, Fengquan Liu, Haiyang Jiang, Guichun Wu, Leiming Wu

**Affiliations:** 1 The National Engineering Laboratory of Crop Resistance Breeding, School of Life Sciences, Anhui Agricultural University, Hefei, 230036 China; 2 Anhui Province Key Laboratory of Crop Integrated Pest Management, Anhui Agricultural University, Hefei, 230036 China; 3 College of Plant Protection, South China Agricultural University, Guangzhou, Guangdong 510624 China; 4 Department of Plant Pathology, College of Agriculture, Guizhou University, Guiyang, 550025 China

**Correction: Stress Biol 6, 3 (2026)**

**https://doi.org/10.1007/s44154-025-00282-1**

Following publication of the original article (Kang et al. 2026), the co-author’s name has been corrected from “Xinhan Hu” to “Qinhan Hu”. The corresponding Chinese character has two distinct Pinyin spellings.

Author Contributions statement has been updated from:

This work was supervised by Leiming Wu. Guichun Wu made contributions to the writing and revision of the manuscript. The original draft was prepared by Yueqi Kang. Mingxiu Ruan, Xinhan Hu, Zihao Gui, Jinyan Zhou and Jianbo Yao collect related publications and revised the manuscript. Yuanyuan Cao, Ting Ding, Bo Wang Fengquan Liu and Haiyang Jiang improved the manuscript. All author(s) read and approved the final manuscript.

To:

This work was supervised by Leiming Wu. Guichun Wu made contributions to the writing and revision of the manuscript. The original draft was prepared by Yueqi Kang. Mingxiu Ruan, Qinhan Hu, Zihao Gui, Jinyan Zhou and Jianbo Yao collect related publications and revised the manuscript. Yuanyuan Cao, Ting Ding, Bo Wang, Fengquan Liu and Haiyang Jiang improved the manuscript. All author(s) read and approved the final manuscript.

The original article (Kang et al. 2026) has been updated.
